# Dorsoventral Patterning in Hemichordates: Insights into Early Chordate Evolution

**DOI:** 10.1371/journal.pbio.0040291

**Published:** 2006-08-22

**Authors:** Christopher J Lowe, Mark Terasaki, Michael Wu, Robert M Freeman, Linda Runft, Kristen Kwan, Saori Haigo, Jochanan Aronowicz, Eric Lander, Chris Gruber, Mark Smith, Marc Kirschner, John Gerhart

**Affiliations:** 1 Department of Organismal Biology and Anatomy, University of Chicago, Chicago, Illinois, United States of America; 2 Department of Cell Biology, University of Connecticut Health Center, Farmington, Connecticut, United States of America; 3 Department of Molecular and Cell Biology, University of California Berkeley, Berkeley, California, United States of America; 4 Department of Systems Biology, Harvard Medical School, Boston, Massachusetts, United States of America; 5 Department of Molecular, Cellular, and Developmental Biology, University of California Santa Barbara, Santa Barbara, California, United States of America; 6 Broad Institute of MIT and Harvard, Cambridge, Massachusetts, United States of America; 7 Department of Biology, Massachusetts Institute of Technology, Cambridge, Massachusetts, United States of America; 8 Express Genomics, Frederick, Maryland, United States of America; Howard Hughes Medical Institute, United States of America

## Abstract

We have compared the dorsoventral development of hemichordates and chordates to deduce the organization of their common ancestor, and hence to identify the evolutionary modifications of the chordate body axis after the lineages split. In the hemichordate embryo, genes encoding bone morphogenetic proteins (Bmp) 2/4 and 5/8, as well as several genes for modulators of Bmp activity, are expressed in a thin stripe of ectoderm on one midline, historically called “dorsal.” On the opposite midline, the genes encoding Chordin and Anti-dorsalizing morphogenetic protein (Admp) are expressed. Thus, we find a Bmp-Chordin developmental axis preceding and underlying the anatomical dorsoventral axis of hemichordates, adding to the evidence from *Drosophila* and chordates that this axis may be at least as ancient as the first bilateral animals. Numerous genes encoding transcription factors and signaling ligands are expressed in the three germ layers of hemichordate embryos in distinct dorsoventral domains, such as *pox neuro, pituitary homeobox, distalless,* and *tbx2/3* on the Bmp side and *netrin, mnx, mox,* and *single-minded* on the Chordin-Admp side. When we expose the embryo to excess Bmp protein, or when we deplete endogenous Bmp by small interfering RNA injections, these expression domains expand or contract, reflecting their activation or repression by Bmp, and the embryos develop as dorsalized or ventralized limit forms. Dorsoventral patterning is independent of anterior/posterior patterning, as in *Drosophila* but not chordates. Unlike both chordates and *Drosophila,* neural gene expression in hemichordates is not repressed by high Bmp levels, consistent with their development of a diffuse rather than centralized nervous system. We suggest that the common ancestor of hemichordates and chordates did not use its Bmp-Chordin axis to segregate epidermal and neural ectoderm but to pattern many other dorsoventral aspects of the germ layers, including neural cell fates within a diffuse nervous system. Accordingly, centralization was added in the chordate line by neural-epidermal segregation, mediated by the pre-existing Bmp-Chordin axis. Finally, since hemichordates develop the mouth on the non-Bmp side, like arthropods but opposite to chordates, the mouth and Bmp-Chordin axis may have rearranged in the chordate line, one relative to the other.

## Abstract 2

Experimental and anatomical evidence for the role of Bmp and Chordin in the development of the dorsal-ventral axis of hemichordates provides insights into the evolution of the chordate lineage.

## Introduction

Arthropods (especially *Drosophila*) and chordates (especially mouse, fish, frogs, and birds) and presumably all bilaterians are fundamentally similar in the development of their body plans [1,2]. Suites of genes are arranged in conserved domain maps in both the anteroposterior and dorsoventral dimensions of developing embryos. Paradoxically, this conservation of axial patterning provides the developmental platform for astonishing anatomical and physiological diversification both within and between phyla. Clearly a major step in the evolution of bilateral animals was the origination of these domain maps and the usage of transcription factors and signaling proteins by which diverse target genes could be expressed at specific times and places in development at subsequent stages.

The evolution of the dorsoventral axis (transverse to the anteroposterior axis) is not well understood. It may have originated during the transition from radial to bilateral animals [3], although recent evidence raises the possibility it was already cryptically present in radial animals [4,5]. Bilateral animals, at least back to the Cambrian, have numerous anatomical and physiological specializations located along this axis, in all three germ layers. The mesodermal tissues such as the heart, blood forming elements (and the direction of blood flow), gonads, visceral muscle, and striated muscle are arranged in dorsoventral patterns. The gill slits (in chordates and hemichordates) and other endodermal organs have similarly conserved patterns in the dorsoventral domain. Ectodermal examples include the central nervous system and epidermal territory. The dorsal and ventral poles of the axis are usually assigned based on the orientation of the animal related to gravity (dorsal up; ventral down) or on the location of the mouth (defining the ventral side). Some of these specializations were presumably added before others in the evolution of bilateral animals. The more widespread the specialization among bilateral animals, the older it is assumed to be. For example, since arthropods and chordates have central nervous systems, and since new molecular data reveal commonalities of their development and organization, the central nervous system has been considered a dorsoventral differentiation already present in the bilateral ancestor [1]. We will address this point in our study of hemichordates.

Preceding the dorsoventral axis of anatomical and physiological specializations in the course of development is a molecular axis. In arthropods and chordates genes for bone morphogenetic protein (Bmp 2,4,7, or Dpp) are expressed in the embryo at one pole and the genes for Bmp antagonists, such as Chordin and Noggin, at the other. All subsequent tissues, organs, and cell types develop in accordance with this Bmp-Chordin axis [1,6]. It may be part of the fundamental body plan of all bilateral animals and may be a key innovation of the bilaterian ancestor (urbilaterian), to which all later dorsoventral differences of anatomy and physiology were attached [1].

Once established in the embryo, two phases of dorsoventral patterning can be distinguished in chordates and *Drosophila* [7]. During the first stage, at pre-gastrula and gastrula stages, ectoderm segregates into two distinct domains, neural and non-neural (i.e., epidermis). Embryonic ectodermal cells that are exposed to high Bmp levels develop as epidermis, whereas cells exposed to low Bmp levels, due to the action of antagonists, develop as neural ectoderm. In both cases, the nervous system develops on the Chordin (low-Bmp) side (called dorsal in the former and ventral in the latter). In vertebrates, a neural default circuitry underlies the decisions, in which Bmp signals repress neural development and promote epidermal development of the ectoderm, whereas elimination of Bmp by antagonists de-represses neural development and represses epidermal [8]. Bmp absence, though necessary, may not suffice for neural development in chordates; fibroblast growth factor may have a licensing role [9]. Likewise, different antagonists of Bmp may operate in different organisms, such as noggin and follistatin in addition to Chordin; the extracellular modulators of Bmp distribution may also vary. This segregation of ectodermal territories along the Bmp-Chordin axis in the first phase accomplishes the centralization of the nervous system in chordates and *Drosophila.*

In the second phase, at post-gastrula stages, Bmp serves to pattern the germ layers in *Drosophila* and chordates [10]. In the vertebrate neural tube there is a new round of *bmp* expression in the roof plate. Hedgehog (Hh) protein and Bmp antagonists emanate from the floorplate and notochord. More than 20 gene expression domains distribute along the dorsoventral axis of the tube in relation to these signals and specify neural cell types [11]. Sensory cells and sensory interneurons (in the dorsal part of the neural tube) arise where there is high Bmp, whereas motor neurons arise in regions of low Bmp and high Hh protein [12,13]. Similar patterning occurs in the *Drosophila* neurogenic ectoderm, where Bmp is opposed by antagonists and by epidermal growth factor [14,15]. In the mesoderm of chordates and *Drosophila,* high Bmp specifies the heart, and visceral muscle, whereas low Bmp (high antagonists) specifies striated body wall muscle [16]. In vertebrates, Bmp levels also specify gut regions and organs of the endoderm [17,18]. The similar anatomical and physiological specializations along the Bmp-Chordin axis in both *Drosophila* and chordates may imply that some of them date back to the bilateral ancestor and were conserved in later divergent lineages of bilaterians. However, it is also possible that some of them evolved independently in different bilaterian lineages within the context of the pre-existing Bmp-Chordin axis. A central question would be whether the segregation of epidermis and neurogenic ectoderm, as a means of centralizing the nervous system, dates back to the bilaterian ancestor or arose independently in several lines of descendents including chordates and arthropods [19].

We report here the study of the origins of the dorsoventral developmental axis in *Saccoglossus kowalevskii,* a member of the hemichordata, a phylum of bilateral adult animals, the sister taxon to echinoderms, and closely related to chordates. These three phyla along with the recently reclassified *Xenoturbella* constitute the deuterostome supertaxon [20]. We expected hemichordate embryos to utilize a Bmp-Chordin axis to pattern their dorsoventral anatomical axis, in light of the conserved use of Bmp-Chordin in dorsoventral patterning of arthropods and chordates. However, hemichordates differ from those phyla in lacking a central nervous system: theirs is diffuse throughout the ectoderm. Nerve cells and epidermal cells are finely intermixed [21]. The epidermis is not broadly segregated from neural ectoderm, and thus seems to lack phase 1 of neural development that is characteristic of arthropods and chordates.

**Figure 1:**
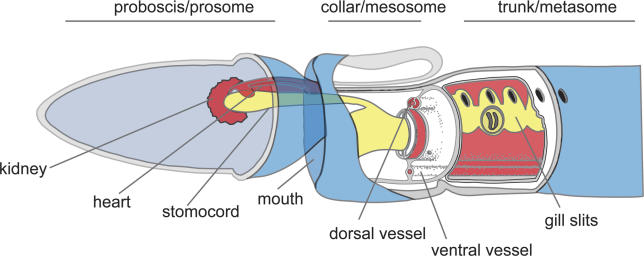
The Anatomy of S. kowalevskii to Illustrate Differentiations along the Dorsoventral Axis The ventral side, assigned because of the mouth location, is down in this schematic figure. Anterior is to the left. Note the three parts of the body: the prosome (proboscis) at the anterior end, the narrow mesosome (collar) in the middle, and the elongated metasome to the posterior (only half the length is shown). Ectodermal derivatives are colored blue, mesodermal red, and endodermal yellow. Prominent structures of the dorsoventral axis include the dorsal and ventral axon tracts, the dorsal and ventral blood vessels, the dorsal stomochord with the heart/kidney complex, the dorsal gill slits, the ventral mouth, and the ventral tail (only in the juvenile, not shown). Redrawn with modification from Benito and Pardos [105].

Yet, despite its diffuse nervous system, hemichordates have a well-developed dorsoventral polarity in all three germ layers, as if phase 2 patterning occurs. On one side (sometimes called dorsal) are localized the gill slits, stomochord, heart/kidney complex, and gonads, as shown in Figure 1. On the other (called ventral) are localized the mouth and post-anal tail [22]. Even for the diffuse nervous system, there are dorsoventral differences in neuronal cell types. The two axon tracts on the opposite midlines differ functionally (the ventral one more associated with motor function). The dorsal tract is internalized in the collar region by a neurulation-like developmental process [23]. Giant neurons develop near the dorsal midline, and a specialized patch of sensory neurons develops near the mouth on the ventral side at the base of the proboscis [21]. Despite these differences, the categorical assignment of dorsal and ventral poles to the hemichordate axis is ambiguous, because the animal lives mostly in a vertical orientation and its mouth is very wide [24]. The mouth side is nonetheless often called ventral, by convention.

Overall, this organization led Nübler-Jung and Arendt [25] to propose that hemichordates have a dorsoventral anatomical axis the inverse of chordates and the same as protostomes such as *Drosophila.* Said in other words, they predicted that the hemichordate mouth falls on the Chordin side of the animal, whereas in chordates it is on the Bmp side. If true, chordates would be unique among bilateral animals in having moved the mouth to the opposite side of the body. A venerable explanation for the chordate anomaly is the inversion hypothesis: an ancestor of the chordate lineage inverted the body dorsoventrally and then moved the mouth to the other side [26]. Although the hypothesis, as usually presented, holds that the ancestor already had a centralized nervous system on one side (ventral in the ancestor, dorsal in chordates after inversion), this is not a necessary provision of the hypothesis. Other germ layers and organs might have had dorsoventral organization at the time of body inversion, and the nervous system might have centralized later.

We have examined the topology of expression of a range of orthologs of genes in S. kowalevskii embryos that are known in vertebrates to depend on the Bmp-Chordin axis for their domain location and to have roles in dorsoventral development. We also investigated the functional importance of Bmp signaling for dorsoventral patterning by RNA interference (RNAi) knockdown and by treatment with ectopic ligand. The comparison of hemichordates to chordates affords insights about which elements of dorsoventral patterning are likely ancestral to deuterostomes and which are unique to chordates or hemichordates. S. kowalevskii indeed expresses many orthologs in a topology that would support the hypothesis of conserved roles in patterning along a Bmp-Chordin axis. Other orthologs, we find, are expressed differently, most notably those that are targets of Hh signaling in the phase 2 patterning of the chordate nervous system. Our functional studies confirm that the Bmp-Chordin axis underpins the dorsoventral anatomical axis of members of this phylum. However, the segregation of neural and epidermal territories does not seem to be a part of this patterning, and this initial phase is not required for other aspects of dorsoventral patterning. Furthermore, dorsoventral patterning seems independent of anteroposterior patterning in *S. kowalevskii,* an independence well known in *Drosophila* but not found in chordates. We discuss the evolutionary implications of our data for the evolution of bilaterians and suggest that the developmental steps involved in centralization of the nervous system (that is, the epidermal-neural segregation) were added independently in different lineages, but always utilizing an existing Bmp/Chordin axis already involved in dorsoventral patterning in the ectoderm.

## Results

Despite the difficulties of assigning definitive dorsal and ventral poles in hemichordates, we will, for descriptive purposes in this section, use the classical definition with ventral represented by the position of the mouth (Figure 1).

### Bmp Signaling and the *bmp* Synexpression Group

**Figure 2:**
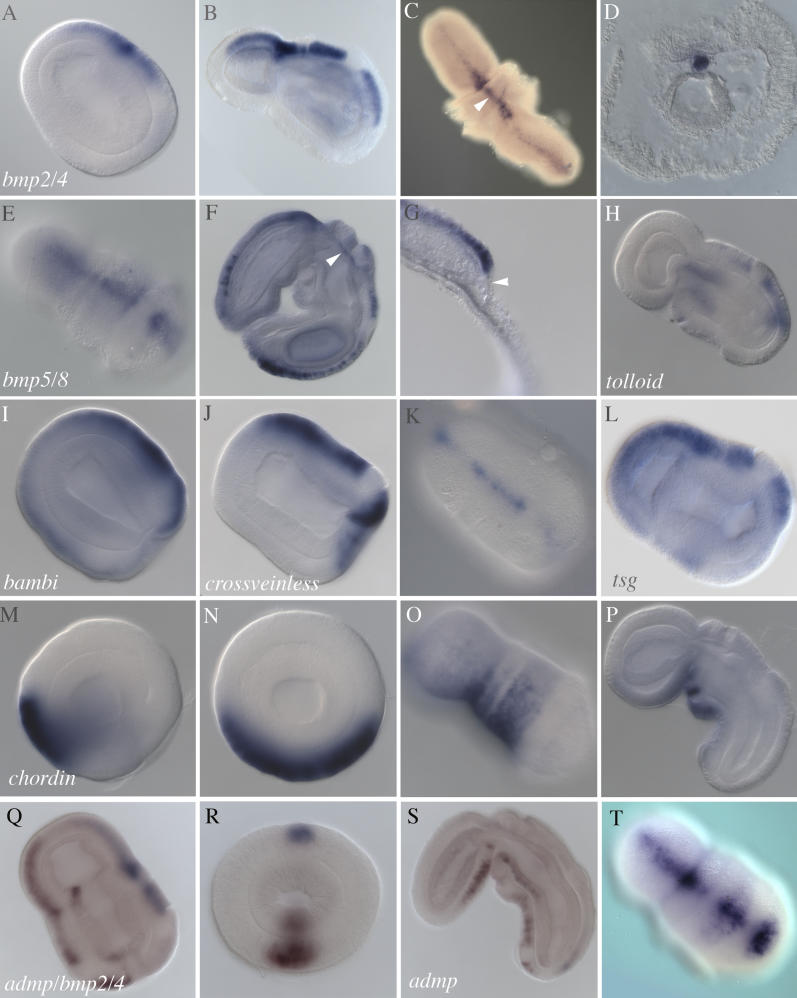
Early Midline Signaling of Bmp and Chordin All embryos are cleared (see Materials and Methods) otherwise specified. All embryos have a similar orientation: anterior in the top left and dorsal in the top right of each panel, unless otherwise specified. (A) Expression of *bmp2/4* at late gastrula stage and (B) at day 3 of development. (C) Hatched juvenile oriented with dorsal toward the viewer. White arrowhead indicates the position of the collar where the *bmp2/4* expression domain follows the submerged ectodermal track of the dorsal cord. (D) Section of a juvenile of a similar stage to (C). Dorsal is at the top of the panel. Note the submerged *bmp* domain, stained purple. (E) Dorsal view of *bmp5/8* expression at day 3 of development with dorsal toward the viewer, and (F) in a hatching juvenile at day 5 of development. White arrowhead indicates the submerged domain in the collar (mesosome) similar to that of *bmp2/4* in panel D. (G) Expression of *bmp5/8* in the far posterior of a late juvenile at day 13 of development. White arrow indicates the position of the anus, before which the *bmp5/8* domain stops. Posterior to the anus is the post anal tail. (H) Expression of *tolloid*/*xolloid* at day 3 of development. (I) Expression of *bambi* at the late gastrula stage. (J) Expression of *crossveinless* at the late gastrula stage and (K) at day 3 of development, with the dorsal midline oriented toward the viewer. (L) *Tsg* expression at day two of development. (M–P) *Chordin* expression from early to late developmental stages. (M) Expression at mid gastrula. (N) Sagittal section an embryo at day 2 of development just anterior to the telotroch; ventral is at the bottom of the panel. (O) Day 3 of development, a surface view of the lateral ectoderm, (P) and day 4 of development, in sagittal section. (Q) *Admp* and *bmp2/4* double in situ hybridization at day 2 of development; brown is *admp* and blue is *bmp2/4*, and (R) a cross section of the same stage just anterior to the telotroch. (S) *Admp* expression at day 5 of development, and (T) surface view of an uncleared embryo, ventral midline toward the viewer.

The site of Bmp signaling in the embryos of chordates and arthropods defines one pole of the Bmp-Chordin developmental axis. Genes whose encoded proteins participate in Bmp signaling, such as *bmps 2, 4,* and *7; tolloid; twisted gastrulation (tsg), crossveinless (cv-2),* and *bambi* [1,27,28], have been grouped in the *bmp4* synexpression group, because they are expressed in the same domain [29]. We have isolated orthologous sequences from S. kowalevskii cDNA libraries. *Bmp2/4* of S. kowalevskii is the ortholog of *Drosophila dpp;* a related ancestral sequence was duplicated in the chordate lineage and diverged to both *bmp2* and *bmp4*. *Bmp5/8* is the S. kowalevskii ortholog of an ancestral gene that duplicated and diverged in the chordate lineage to *bmps 5,6, 7,* and *8* and in the *Drosophila* lineage to *screw* and *glass bottom boat* [30]. Expression results are shown in Figure 2.

In *S. kowalevskii, bmp2/4* expression begins at gastrulation in the ectoderm on one side (Figure 2A). As development proceeds, it continues in a sector and eventually narrows by day 3 to a thin stripe on the dorsal midline of the ectoderm, from the apical organ to the anus (Figure 2B and 2C). As dorsal ectoderm of the collar rolls inward on day 3 (“neurulation”), *bmp* expression comes to lie in the submerged ectoderm of the collar axon tract (Figure 2D). In juveniles with 2–3 gill slits (14 d post fertilization), expression continues in a tight midline reaching to the anus, but not extending into the post-anal tail (unpublished data). The expression of *bmp5/8* closely resembles that of *bmp2/4* (Figure 2E–2G); it eventually narrows to a stripe on the dorsal midline and is excluded from the post-anal tail (Figure 2F and 2G). Xolloid is a metalloprotease that in chordates cleaves the complex of Bmp with Chordin, releasing Bmp to bind to its receptors [31,32]. In *Drosophila,* the orthologous protease, Tolloid, accomplishes cleavage of the Dpp/Sog complex, to similar ends. The proteins are essential for active Bmp/Dpp signaling. In S. kowalevskii the expression of *tolloid* begins after gastrulation in the posterior dorsal region of the ectoderm (unpublished data). By day 2 of development, it continues on the posterior dorsal midline in the ectoderm, but anteriorly only to the boundary of the metasome and mesosome. Expression is also detected at this stage in the prospective pharyngeal endoderm, and in a small patch of posterior dorsal endoderm (Figure 2H). *Bambi,* which encodes an inhibitory pseudoreceptor of Bmp that is co-expressed with *bmp4* in *Xenopus* and mouse [33,34], is similarly co-expressed in *S. kowalevskii,* on the dorsal midline shortly after gastrulation (Figure 2I). Low levels of expression are also detected throughout the ectoderm. In later stages, its dorsal expression declines to undetectable levels (unpublished data). Tsg, a modifier of Bmp signaling, binds to Chordin and Tolloid to agonize or antagonize Bmp signaling, depending on the presence of yet other modifiers [35–37]. In *S. kowalevskii, tsg* is expressed on the dorsal midline (Figure 2L) at late gastrulation, and at low levels throughout the ectoderm. Like *bambi,* its dorsal expression declines after day 2 of development to undetectable levels. The final member of the *bmp4* synexpression group in this study is *cv-2, a crossveinless* ortholog. Originally cloned from *Drosophila melanogaster,* it is expressed in regions of high Bmp signaling in the metamorphosing wing, but not during embryonic development [38]. In chordates it is expressed in regions of high Bmp signaling, such as the roofplate of the neural tube, and its encoded protein modulates Bmp signaling [39–41]. In *S. kowalevskii, cv-2* is expressed on the dorsal midline (Figure 2J) of the embryo. Like *bambi* and *tsg,* its expression diminishes in later stages. To conclude, five members of the *bmp4* synexpression group, originally identified in D. melanogaster and vertebrates, show dorsal midline domains of expression in S. kowalevskii. We thus find significant correlative evidence of Bmp signaling from this midline in hemichordates. In a later section of results, we will experimentally investigate the role of Bmp signaling in hemichordate dorsoventral patterning.

We also cloned an ortholog encoding Chordin, a Bmp antagonist, from S. kowalevskii and examined its expression in relation to the *bmp* domain. Expression begins in a sector of ectoderm of the early gastrula, the presumed prospective ventral side, since it is opposite the *bmp2/4* domain as shown by double in situ hybridization (see thumbnail photo). At early stages, its breadth of expression extends almost to the dorsal side in the mid-level ectoderm (Figure 2M–2P), though less extensively in the anterior and posterior ectoderm. At later stages, *chordin* expression remains broad, strongest in the mesosome and anterior metasome, and tapering off at the telotroch (a dense ciliated band) in the posterior metasome. Anteriorly, *chordin* is expressed in the ectoderm of the posterior prosome, tapering off toward the tip (Figure 2O). By day 4, its expression shrinks to a small ventral patch in the anterior metasome, mesosome, and posterior prosome ectoderm (Figure 2P). This pattern of expression of *chordin,* centered on the ventral midline, strongly suggests that its encoded protein and Bmp on the dorsal side are involved in an antagonistic interaction, as found in other bilateral animals. In S. kowalevskii as well as *Drosophila, chordin* is only expressed in the ectoderm, whereas in *Xenopus,* it is expressed in both the ectoderm and mesoderm [42]. In the course of development of S. kowalevskii to the hatched juvenile stages, the only domains of expression of *chordin* and *bmp* are the midline stripes described above; there are no secondary domains.

Another gene from the Bmp sub-family of transforming growth factor-beta signaling molecules, which encodes a protein with a key role in vertebrate organizer function, is *anti-dorsalizing morphogenetic protein (admp).* No *admp* ortholog has been found in *Drosophila.* Like Bmp2, 4, and 7, the Admp protein may bind to Chordin and be rendered inactive in signaling until the Tolloid protease releases it [43]. Unlike the genes of other Bmps, *admp* appears to be co-expressed with *chordin,* not opposite it, and to be repressed by Bmps. According to current views, Admp acts like a Bmp signal in concert with the other Bmps, when released on their side of the embryo [44]. We cloned the S. kowalevskii ortholog of *admp* from an expressed sequence tag collection. The ortholog is expressed on the ventral midline of embryonic ectoderm and also in some regions of ventral midline endoderm (Figure 2Q–2T). Expression begins in the ventral ectoderm and endoderm of the late gastrula (unpublished data). Thereafter, as the embryo elongates, ectodermal *admp* expression extends in a thin line from the anterior tip to the anus, interrupted only by the telotroch (Figure 2Q). It is expressed directly opposite *bmp2/4* as shown in Figure 2R. At day 4, *admp* is still expressed along the ventral midline of the embryo; however, its level is much lower in the mesosome and anterior prosome (Figure 2S). Ventral endoderm expression of *admp* is most prominent in two domains; the more anterior of these in the metasome becomes more intense than the other by day 4 of development (Figure 2S). Thus, as in chordates, *chordin* and *admp* are co-expressed in hemichordates on the side opposite Bmps. Unlike chordates, the hemichordate expression is in the ectoderm and endoderm, rather than in the mesoderm. Thus like *chordin* expression, *admp* does not identify organizer-like mesoderm in hemichordates.

### Dorsally Restricted Domains of Target Gene Expression

**Figure 3:**
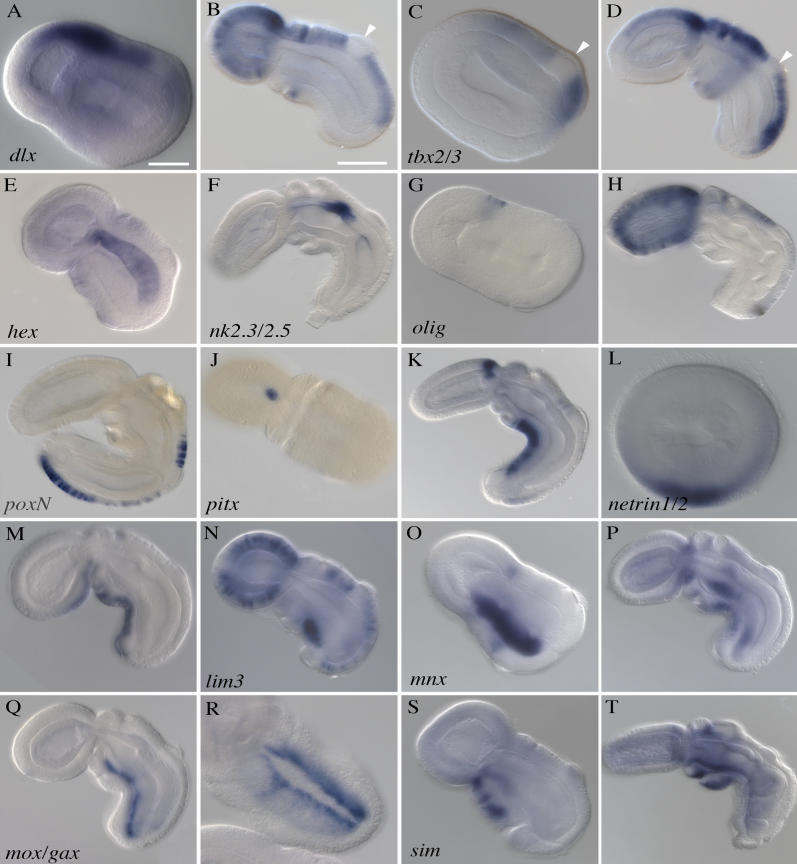
Genes Expressed with Dorsal or Ventral Domains These genes were chosen as candidate targets of Bmp activation or repression. All embryos are oriented as in Figure 2 with anterior to the top and left of each panel and dorsal in the top right of each panel, unless otherwise specified. The telotroch or ciliated band is marked by white arrowheads. Expression of *dlx* at (A) day 2 of development, just after gastrulation and (B) at day 3 of development. (C) *Tbx 2/3* expression just after gastrulation, and (D) at day 3 of development. (E) Expression of *hex* at day 3 of development (F) Expression of *nk2.3/2.5* at day 4. (G) Expression of olig on day 2, just after gastrulation, and (H) at day 3. (I) *poxN* expression at day 4 of development. (J) *Pitx* expression at day 2 of development, dorsal midline toward the viewer, a glancing optical section through the dorsal-most ectoderm. (K) *Pitx* expression at day 4 of development. Note the two domains of expression. (L) *Netrin* expression, a transverse section of a post-gastrula embryo at the level of the ciliated band. Note the broad ventral expression of *netrin*, and (M) the more narrow domain at day 3 of development. (N) Expression of *lim3* at day 3 of development. (O) Expression of *mnx* at day 2 of development, and (P) at day 4. Note the ventral endodermal expression. (Q) Expression of *mox* (also called *gax*) at day 3 of development, and (R) a close up of the ventral domain at day 3, ventral midline toward the viewer, displaying the metasome and part of the mesosome. (S) Expression of *sim* at day 2 of development, and (T) at day 5.

Orthologs encoding seven transcription factors exhibit dorsal domains of expression in the different germ layers, providing evidence of a dorsoventral pattern of transcriptional regulation in hemichordates, previously unreported. These genes, which include *distalless (dlx), tbx2/3, olig1/2, pox neuro (poxN), pituitary homeobox (pitx), hex,* and *nk2.3/2.5,* are candidate targets of Bmp activation. Expression results are shown in Figure 3. In a later section of Results, we provide evidence that their domains indeed expand and contract when Bmp signaling is manipulated, but first we must describe their domains in normal development.

In the ectoderm, *dlx* is expressed in a stripe on the dorsal midline, like *bmp2/4* (Figure 3A). Also, it is expressed in a uniform speckled pattern in the prosome ectoderm, as reported earlier [45]. Its expression begins after gastrulation in a sector of ectoderm, the prospective dorsal midline (Figure 3A), and continues throughout all stages examined, closely resembling *bmp2/4* expression. Like *bmp2/4* and *bmp5/8,* its domain is submerged in the anterior mesosome, where the dorsal axon tract is internalized (unpublished data). In vertebrates, *dlx* paralogs have multiple developmental roles, but all share a role in patterning neural crest cells on the dorsal midline where the neural folds meet, a site of high Bmp levels [46,47].

Similar expression is found for the S. kowalevskii T-box gene, *tbx2/3.* In vertebrates, *tbx2* and *tbx3* are distinct from other T-box genes by being transcriptional repressors rather than activators [48]. Although *tbx2* is expressed in several domains during chordate development, it is expressed in neural crest cells forming the sensory dorsal root ganglia, common to all vertebrate species investigated [49,50]. Interestingly, *bmp2* regulates *tbx2* and *tbx3* in early cardiac development in vertebrates [51], and in sea urchins *tbx2/3* responds to Bmp signaling in the establishment of the oral/aboral axis [52]. In *Drosophila,* the ortholog of *tbx2/3, optomotor blind,* is also regulated by Dpp during wing disk development [53]. In *S. kowalevskii,* expression of *tbx2/3* begins during gastrulation in a sector of the ectoderm and rapidly restricts down to a dorsal stripe, with its strongest expression posterior to the telotroch ciliated band. Its midline domain is broader than found for *bmp2/4* and *dlx* (Figure 3C and 3D). In the anterior metasome near the first gill slit, ectodermal expression extends ventrally and encircles the embryo as a band.

Three further genes, *olig, pox neuro,* and *pitx* exhibit dorsal midline expression in sub-regions of the embryo's length. In vertebrates, *olig1* and *2* are expressed in the ventral spinal cord in the region that gives rise to oligodendrocytes and motor neurons, whereas *olig 3* is expressed in the dorsal cord [54]. Bmp and sonic hedgehog (Shh) have been implicated in their activation in these regions [55]. We see the initial expression of the *olig* ortholog of S. kowalevskii in a dorsal spot at the base of the prosome, in a cluster of cells (Figure 3G). Later, the expression expands to the entire proboscis ectoderm and to a dorsal midline of spots of cells along the body length (Figure 3H).

Pox neuro is a *pax* gene of *Drosophila* and other arthropods, closely related to the *pax 2/5/8* group of genes and the cnidarian *paxA* group [56], but there is no clear ortholog in vertebrates. The PoxN protein plays a role in both the peripheral and central nervous systems of *Drosophila* and may be involved in determining neuroblast identity [57]. We have cloned five *pax* genes from *S. kowalevskii,* two previously published in Lowe et al. [45]. In cladograms, *poxN* clusters with Pax A and C of cnidarians and *poxN* of *Drosophila;* it is distinct from the *pax 2/5/8* group of genes (Figure S-2H). In S. kowalevskii its expression begins on day 3, quite late in development, after the three major body divisions have been established. It is detected in isolated cells of a tight line of expression on the dorsal midline of the metasome (Figure 3I).

*Pitx* plays multiple roles in chordate development. Though named for its role in pituitary development [58], it also participates with *nodal* in establishing deuterostome left/right asymmetries [59] and in ventral posterior patterning [60]. In *S. kowalevskii, pitx* is expressed in two widely separated domains. One appears at gastrulation in the posterior ventral ectoderm in a broad band of scattered cells, just anterior to the telotroch (unpublished data). In later stages, expression narrows to a stripe along the ventral midline (Figure 3K). A second domain of strong expression appears at day two of development, as the three body regions begin to resolve, at the base of the proboscis on the dorsal midline (Figure 3J). This distinct spot is approximately at the site where the proboscis pore later forms, connecting the anterior coelom to the exterior, just left of the dorsal midline. During those later stages, as the stomochord projects into the prosome directly underneath this spot, *pitx* expression extends into the mesoderm of the heart/kidney complex, just dorsal to the stomochord. It remains unknown whether this prosome domain has any relation to an ancestral pituitary rudiment predicted by Goodrich [61] to occupy this location.

Three transcription factors show dorsal domains in the endoderm. The first is an *nk* homeobox gene, we have named *nk-2.3/2.5* that is related to *Drosophila tinman, amphink-tin,* and a range of *nk2* class vertebrate genes that all play roles in cardiac development (see gene tree in S-4P). We have succeeded in cloning only one member of this related group of vertebrate genes, as was also reported in amphioxus [62], suggesting that the vertebrate *nk2* class cardiac genes represent a diversification of an ancestral copy of a *tinman-*related gene in basal chordates. We do not detect expression of *nk-2.3/2.5* until relatively late in development (day 4) in the most dorsal part of the pharyngeal endoderm, between the first pair of gill pouches as they begin to develop (Figure 3F). A similar domain of expression is detected ventrally in the floor of the developing pharyngeal endoderm of amphioxus [62]. *Tinman* and related genes are best known for their conserved roles in the development of cardiac mesoderm [63]. However at no point in development do we detect expression of *nk 2.3/2.5* of S. kowalevskii in the structure classically described as the heart in the axial complex, at the tip of the stomochord [64]. Moreover, it is not expressed in mesoderm, only dorsal midline endoderm, at the stages we have examined.

The second gene detected in the dorsal endoderm is the transcriptional repressor *hex.* In vertebrates it is expressed in the anterior visceral endoderm and plays an early role in blocking mesoderm induction and favoring anterior neural development; later it plays a role in the development of the liver and the thyroid [65,66]. Expression of *hex* in S. kowalevskii begins very early in the gastrula and shows a tight restriction to the prospective dorsal endoderm in a region of expression directly beneath the *bmp2/4* stripe (unpublished data). Expression continues in the dorsal endoderm in the post-gastrula from the very posterior endoderm to the most anterior endoderm except for the mouth (Figure 3E). In later developmental stages, the expression becomes thinner and more tightly associated with the dorsal midline. The expression in the anterior is stronger than more posteriorly (unpublished data). The final gene expressed in the dorsal endoderm is *pax1/9.* As we reported earlier [45], it is expressed during the formation of the gill slits.

To conclude, we report seven new genes with dorsal domains of expression in hemichordates. These seven were not difficult to find, and we expect that many more genes exhibit such dorsoventral expression, perhaps all dependent on the Bmp-Chordin axis.

### Ventrally Restricted Domains of Target Gene Expression

Orthologs encoding a signaling ligand (Netrin) and four transcription factors (Mnx, Lim3, Mox, and single-minded [Sim]) are expressed in distinct ventral domains in the three germ layers of S. kowalevskii embryos, and hence they are candidate targets of Bmp repression. To these should be added the ventral domain of *pitx* expression, described above. In a later section of Results, we will provide evidence that these genes are indeed Bmp targets, since their domains are expanded and contracted when Bmp signaling is manipulated, but first we must describe the normal domains.

Comparative studies of *netrin* show that the encoded protein plays a conserved role in axonal guidance [67,68]. In *Drosophila* the two *netrin* genes, A and B, are expressed along the ventral midline, and the proteins are signals for axon repulsion and attraction by commissural neurons in short and long range signaling [69]. In vertebrates, an ancestral *netrin* gene underwent a series of duplications and diversifications to at least four *netrin* genes. In the central nervous system, *netrin 1* is expressed in the floorplate and *netrin 2* in the ventral third of the developing spinal cord and floorplate [68,70]. As in *Drosophila* the proteins elicit either commissural axon attraction or repulsion depending on the receptor expressed by the responding growth cone. Gene tree analysis shows that our *netrin* from S. kowalevskii groups with the *netrin 1* and *2* classes of genes (Figure S3-K). Lower deuterostomes probably have only one gene of the *netrin1/2 class* [71]. Expression of *netrin 1*/*2* begins at late gastrulation in a small spot in the posterior ventral ectoderm (unpublished data). After gastrulation, the domain extends from just behind the telotroch forward to the anterior proboscis, though not to the apical tip region (Figure 3M). When viewed in section, the early expression is strongest at the ventral midline, diminishing laterally (Figure 3L). In mid-stage embryos (day 4), its expression is uneven along the ventral midline, forming patches with large gaps at the telotroch and in the mesosome (unpublished data). In later stages the expression narrows to a ventral midline stripe extending from the telotroch to a point midway into the proboscis (Figure 3M and unpublished data). After hatching, it becomes restricted to a very thin line on the ventral midline under the ventral axon tract, extending into the tail. This expression is consistent with Netrin's possible involvement in S. kowalevskii in attracting some axons from the general ectoderm into the ventral axon tract and perhaps repelling others into the dorsal axon tract.

The homeobox gene *mnx* has been implicated in the development of motor neurons in *Caenorhabditis elegans, Drosophila,* and chordates [72]. It is expressed in neurogenic regions, such as the ventral-lateral neural tube of chordates, and its encoded transcription factor may play a conserved role in motor neuron development [73]. The *mnx* ortholog of S. kowalevskii has an initial expression profile similar to that found in amphioxus; that is, in the endoderm of post-gastrula embryos [73] rather than in neurogenic ectoderm. In amphioxus, this endoderm expression is restricted dorsally, whereas in hemichordates it is restricted ventrally in posterior endoderm (Figure 3O). Two small patches of expression appear in ventral ectoderm of the metasome, just anterior to the telotroch, and in dorsal ectoderm at the prosome-mesosome boundary. In later developmental stages (day 3), the expression in the endoderm splits into two domains, an anterior one in the ventral pharynx and a posterior one in the ventral endoderm (prospective gut). Ectodermal expression is eventually detected in a ventral patch of the metasome anterior to the telotroch, and in a thin ring of expression at the prosome-mesosome boundary (Figure 3P). Although the endoderm expression of *mnx* in hemichordates is not understood, it should be noted that hemichordates (like echinoderms) are reported to have an endodermal nervous system [21].

Some overlap of *mnx* expression is detected with another homeobox gene implicated in motor neuron patterning, *lim3* [74]. *Lim3* is expressed in the same ventral population of ectodermal cells as *mnx* and *pitx,* and no expression of it is found in endoderm (Figure 3N). It is also expressed in the developing prosome ectoderm. Whether these domains in the ectoderm are really associated with the specification of motorneurons is unknown; currently no information is available on neuronal cell type specification in this animal. However, the ventral axon tract is more associated with motor function, and the body wall musculature of the trunk is primarily ventral [75].

*Sim* is another candidate Bmp target from *S. kowalevskii.* Its expression is known in D. melanogaster and chordates. In *Drosophila, sim* is considered a “master control gene” in the development of ventral midline mesectodermal cells [76], with significance for dorsoventral patterning of the nervous system. In chordates it has somewhat variable expression, including in the notochord of amphioxus [77], the axial mesoderm, and the ventral sector of the neural tube in vertebrates [78,79]. The evidence for a conserved role of the encoded protein in *Drosophila* and chordates remains weak. In hemichordates, it is expressed in the ventral endoderm of the mesosome and anterior metasome back to the first gill slit and in ventral ectoderm of the same region (Figure 3S and 3T). Although it is expressed on the ventral midline, further analysis of *sim* in S. kowalevskii is needed before it can be ascribed a role in the establishment of this midline.

Finally, the only restricted marker of mesoderm that we have detected in this study is *mox* (also called *gax*). This gene is a member of the extended *hox* complex and may play a conserved role in mesoderm development throughout chordates and lophotrochozoans, though absent in ecdysozoans such as in C. elegans and *Drosophila* [80,81]. In vertebrates, *mox* is expressed only in trunk mesoderm, not head mesoderm. In *S. kowalevskii, mox* expression begins post gastrulation after the coeloms have pouched out of the archenteron by enterocoely. Expression is initially detected exclusively in the paired coeloms of the metasome, with no obvious dorsoventral asymmetry (unpublished data). As development continues, expression in the dorsal-most region of the metacoels is down-regulated. Ventral expression continues until only a thin line of *mox* expression remains in the developing metasomal mesoderm and extends forward into the mesocoels at later stages (Figure 3Q and 3R).

In summary, we have obtained seven genes with distinctive dorsal expression domains (to which the previously reported *pax1/9* should be added) and five with ventral domains, all of which are candidates for activation or repression by Bmp of the Bmp-Chordin axis of hemichordates.

### Mechanisms of Neural Tube Dorsoventral Patterning: Are They Conserved in Hemichordates?

Three genes were chosen for study in S. kowalevskii because of their well-known roles in dorsoventral patterning of the chordate neural tube: *shh, nk2.2,* and *msx. Shh* is released from the floorplate of the neural tube, opposite Bmp from the roofplate, and it activates a suite of genes in the ventral sectors of the neural tube [82], whose encoded transcription factors specify different kinds of ventral neurons. *Nk2.2* is one such gene; it is activated at high *Shh* levels found near the floorplate [83]. *Msx* on the other hand is expressed in dorsal regions of the neural tube in response to Bmp signals from the roof plate and nearby epidermis, and its encoded transcription factor specifies sensory interneurons [84]. The relative placement of *netrin, nk2.2, gsx,* and *msx* domains in the chordate neural tube is similar to the placement of the expression domains of orthologous genes in the *Drosophila* nerve cord. This similarity has been taken as evidence for the homology of the centralized nerve cords of the two groups [85]. Countering the hypothesis of homology is the evidence that many other genes are expressed differently in the two cords and that, while *Shh* is the counter-posed signal to Bmp in the chordate case, epidermal growth factor may have that role in arthropods.

**Figure 4:**
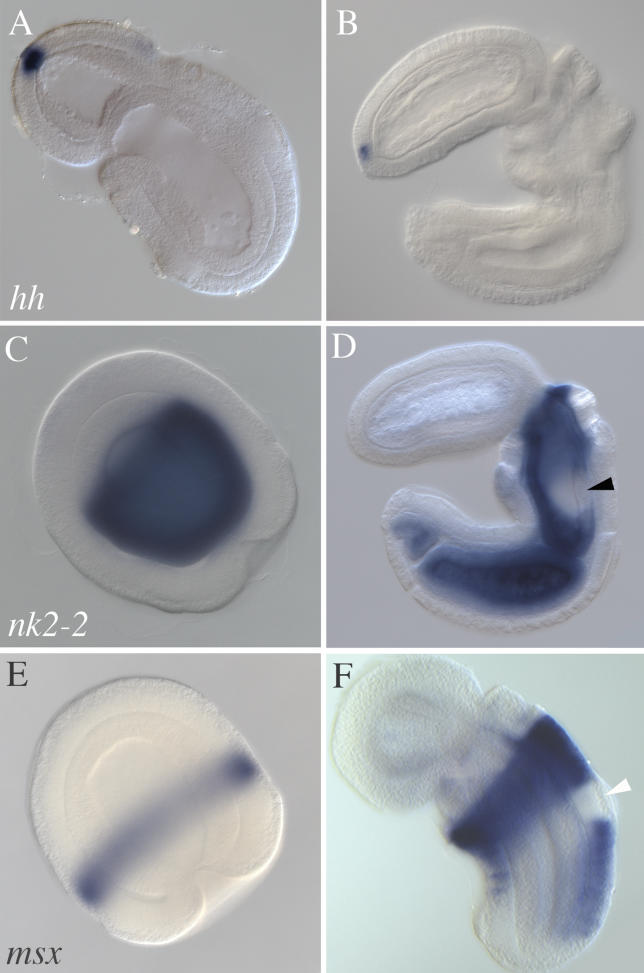
Expression in S. kowalevskii of Orthologs of Chordate Genes Important in Dorsoventral Patterning of the Neural Tube All embryos are shown as optical sections, and oriented in a similar manner as in Figure 2 with anterior to the top and left of each panel and dorsal in the top right of each panel, unless otherwise specified. (A) *Hh* expression in the apical tip of a day 3 embryo, and (B) at day 5 at the same location. (C) Endodermal expression of *nk2–2* (also called *nkx2.2*) in the late gastrula, with a sharp delineation between presumptive proboscis mesoderm and definitive endoderm, and (D) at day 4, with expression continuing in the endoderm but down-regulated in the pouches of the forming gill slits (black arrowhead). (E) Expression of *msx* in the late gastrula, and (F) at day 3. Expression occurs exclusively in the ectoderm of the metasome and is down-regulated in the telotroch, the cilated band, as marked (white arrowhead).

In *S. kowalevskii,* we find that the expression profiles of the *hh, nk2.2,* and *msx* orthologs do not at all resemble the expression domains of vertebrates or *Drosophila,* as shown in Figure 4. We could isolate only one kind of hedgehog ortholog from *S. kowalevskii,* but this may exhaust the hemichordate repertoire since basal deuterostomes such as amphioxus and sea urchins have but one gene [86,87], whereas vertebrates have up to four paralogs of *hh* [86]. The expression of *hh* in S. kowalevskii begins at day 2, in a small patch at the apical tip of the prosome ectoderm (Figure 4A), and it continues in the same domain throughout all stages examined (Figure 4B). Low level expression also occurs in the anterior gut (unpublished data). At no time is *hh* expressed in a dorsal or ventral midline domain, for example, close to the *netrin* ventral domain. The *nk2. 2* of S. kowalevskii was cloned by low stringency hybridization. Its expression begins at gastrulation in the endoderm (Figure 4C) rather than ectoderm, as has been also found in amphioxus but not other chordates [88]. Its domain in S. kowalevskii clearly delineates endoderm from the anterior mesendoderm that becomes the prosome mesoderm. It is continuously expressed throughout the endoderm until hatching; however, it is down-regulated in the dorsal endoderm at the site where gill slits form on day 3, and thereafter (Figure 4D). As the stomodeum (mouth) opens at day 2–3 of development, the expression of *nk2.2* extends into the ectoderm in two narrow wings near the mouth, leading toward but not reaching the dorsal midline (Figure 4D). No other ectodermal domain was found during development. Just as *hh* is not expressed in a chordate-like dorsoventral domain, the *nk2.2* gene, which in chordates depends on Hh signaling for expression, also reveals no chordate-like domain.

*Msx* was cloned from S. kowalevskii cDNA libraries by low stringency hybridization using a probe based on the *msx* homeodomain of *Strongylocentrotus purpuratus*. Expression begins during gastrulation as a circumferential ectodermal stripe in the prospective anterior metasome (Figure 4E). Its early ring-like domain resembles that of *engrailed* [45] except that the ring of the latter is interrupted on the dorsal midline. At later developmental stages, as dorsal flexure occurs, the *msx* domain expands posteriorly into the dorsal metasome (Figure 4F). The highest expression occurs just anterior to the telotroch ciliated band.

Of these three genes, all of which have conserved roles in dorsoventral neural patterning in chordates, none shows evidence of such a role in the hemichordate *S. kowalevskii*. The similar domains and specification roles of *msx* and *nk2.2* along with *gsx* (which we have not yet obtained) in the central nervous systems of vertebrates and *Drosophila* have served as evidence of the homology, ancestry, and conservation of central nervous systems across all bilaterians. Since we find no such correspondence in *S. kowalevskii;* either the domains and roles were lost in the lineage leading to hemichordates (and perhaps echinoderms), or they were independently co-opted in arthropods and chordates.

### Functional Effects of Bmp Signaling

**Figure 5:**
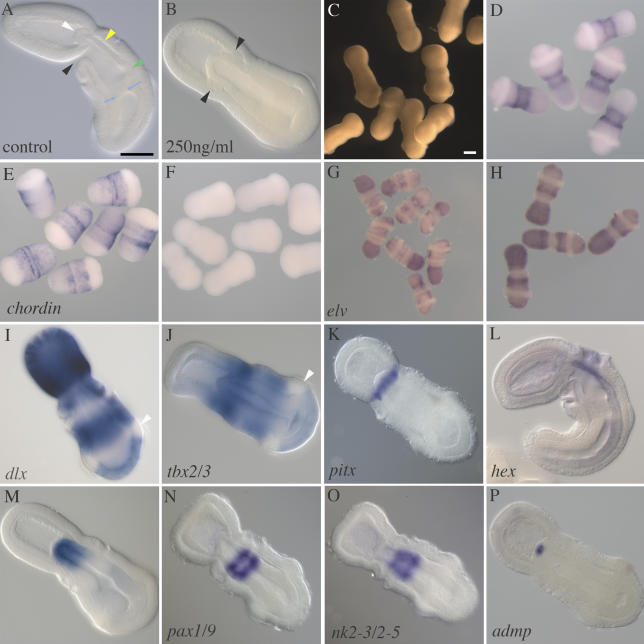
Dorsalization of the S. kowalevskii Embryo by Application of Exogenous Bmp4 Protein Exogenous Bmp4 protein was applied to the late blastula embryo (14 h post fertilization), and development was allowed to continue. All embryos are shown at a similar developmental stage, day 3 of development. (A) Side view of a control embryo cultured without Bmp4. The mouth is indicated by a black arrowhead on the ventral side. The normally developing endoderm shows a dorsal, anterior projection of the gut called the stomochord, indicated with a white arrowhead. One of the mesocoels is clearly visible on the dorsal midline, indicated by a yellow arrowhead. The endoderm is divided into two sections, the pharyngeal region in the anterior, divided from the posterior gut region by a posterior constriction shown by blue arrows. The first gills slit is indicated by a green arrowhead, just anterior to the gut division. (B) Embryos treated with 250ng/ml of Bmp 4, fixed at the same time as the control in panel A. The dorsoventral orientation is not possible to determine since they are cylindrically symmetric. Black arrowheads indicate thick condensations of mesenchyme around the anterior gut. (C) Embryos fixed at a similar development stage following a treatment with 500 ng/ml Bmp4 displaying a consistent phenotype between samples. Note the flattened anterior end and the thick connection of prosome and mesosome. (D) Expression of *bmp2/4* following treatment with Bmp4 protein showing activation of endogenous expression throughout the ectoderm. (E) Stereomicrographs of uncleared embryos showing the expression of *chordin* in control embryos, with broad ventral expression, and (F) embryos following treatment with Bmp4 protein at 100 ng/ml. (G) Stereomicrographs of uncleared embryos showing the expression of *elv* in control embryos, with broad expression, but stronger at the midlines, and (H) embryos following treatment with Bmp4 protein at 100 ng/ml. Note the persistence of *elv* expression; it is not repressed by Bmp4. (I) Ubiquitous ectodermal expression of *dlx* following treatment with 100 ng/ml of Bmp4. (J) Expression of *tbx2/3* expands throughout the ectoderm following treatment of the embryo with Bmp4 protein (250 ng/ml). White arrowheads indicate the position of the telotroch/ciliated band. (K) Expression of *pitx* expands from a spot to a ring around the base of the prosome in both the ectoderm and underlying mesenchyme, after Bmp4 protein treatment. (L) Control expression of *hex* at day 4 of development, and (M) following treatment with Bmp4 at 100ng/ml. (N) *Pax1/9* expression expands from a dorsolateral spot to a circumferential ring in the endoderm following Bmp4 treatment at 100 ng/ml. (O) Like *pax1/9,* the *nk2–3/2–5* domain expands from a short dorsal stripe to a ring in the endoderm, after Bmp4 treatment. (P) Expression of *admp* in the most anterior endoderm following treatment with 500 ng/ml of Bmp4. This is a residual spot (thus showing that the staining procedure has worked), whereas the entire ventral domains of ectoderm and endoderm have disappeared.

**Figure 6:**
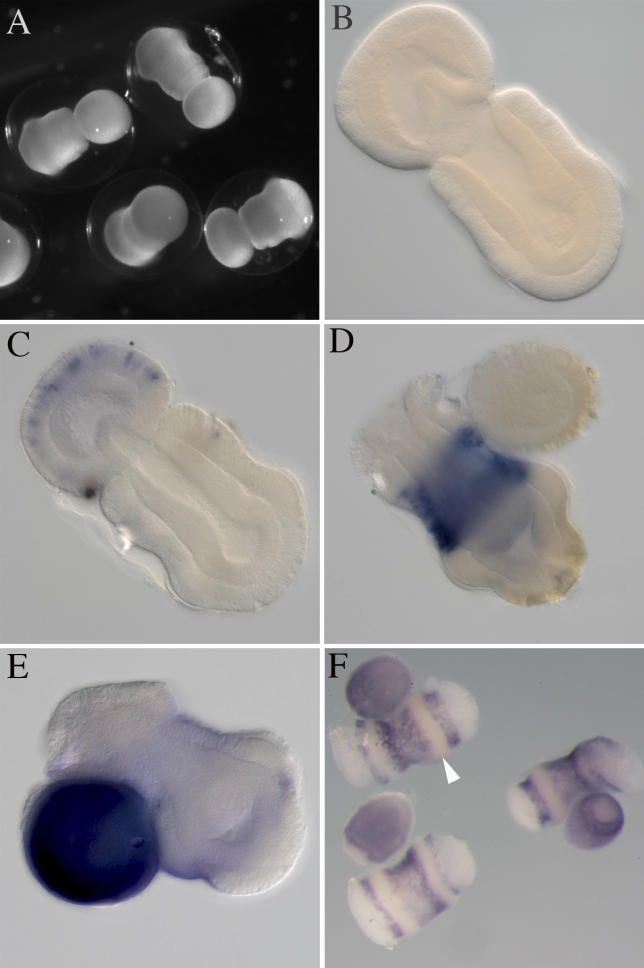
Ventralization of the S. kowalevskii Embryo following Injection of the Egg with Anti-*bmp* siRNAs Eggs were injected immediately following fertilization with *bmp* siRNA and development was allowed to continue. (A) Low magnification micrograph of a group of embryos at the beginning of day 3 of development from injected eggs, showing the consistency of the phenotype. The dorsoventral orientation cannot be determined since the embryos are cylindrically symmetric. Note the deep indentation between the prosome and mesosome, and the short posterior end beyond the telotroch (ciliated band). (B) Optical section of an embryo at the same stage of development from an injected egg. (C) In the siRNA treated embryo, the expression of *dlx* continues in scattered cells in the anterior ectoderm of the prosome but disappears from the dorsal midline. (D) Expression of *pitx* at day 4 of development in a siRNA treated embryo, showing expanded expression throughout the majority of the metasome anterior to the telotroch, whereas the prosome dorsal spot is absent. The prosome has detached from the mesosome at this stage, as the mouth indentation encircles the embryo. The same developmental stage is shown in panel E and F. (E) Expression of *admp* expands strongly throughout the ectoderm of the detached prosome. (F) Low magnification micrograph of day 4 embryos showing expansion of expression of *netrin* from a ventral midline stripe to the entire ectoderm including that of the detached prosome. White arrowhead shows the position of the telotroch.

To test the importance of the Bmp-Chordin axis for the development of the definitive dorsoventral axis of *S. kowalevskii,* we exposed embryos globally to Bmp protein, or we depleted the embryo of Bmp using RNAi. To score the effects, we used most of the 13 genes we now know to be expressed in dorsal or ventral domains in *S. kowalevskii,* as well as features of the overall anatomy of the treated embryos. Of particular interest is the development of the nervous system in treated embryos, which is normally diffuse throughout the ectoderm even though the embryo possesses a Bmp-Chordin axis. Embryos and expression results are shown in Figures 5 and 6, and Table 1 summarizes the results of the following two sections.

**Table 1:**
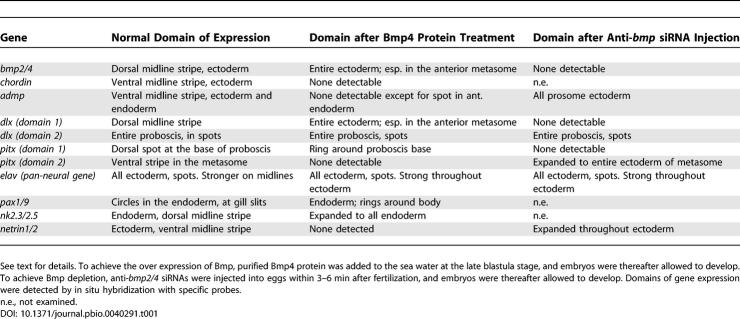
Bmp Levels Control Dorsoventral Domains of Gene Expression in S. kowalevskii

### Over-Expression by Exposure to Exogenous Bmp Protein

When Bmp from vertebrates was applied directly to ascidian embryos, it had the same over expression effects as injection of mRNA for Bmp [89]. We therefore added zebrafish recombinant Bmp 4 protein to seawater at a range of concentrations to assess its effects on the development of S. kowalevskii embryos. All treatments significantly altered development (Figure 5B and 5C). From a morphological and molecular perspective, the embryos are “dorsalized,” in a dose dependent manner (unpublished data). Treated embryos are cylindrical in shape, more elongated than normal, flattened at the proboscis tip, and lacking the dorsal flexure that normally results from asymmetric tissue extension. The mouth is absent, and instead of a thin neck, the mesosome is thickly connected to the prosome. Also the first gill slits are entirely lacking, although at very low levels of Bmp exposure (10 ng/ml) a deep circumferential groove forms in the ectoderm at the position along the anteroposterior axis at which the gill slits would first perforate. The organization of the anteroposterior axis is generally unaffected.

To supplement the morphological observations, we investigated the Bmp dependence of the expression of various genes in the three germ layers. First we assayed the expression of *bmp2/4* itself and found that this mRNA is elevated to detectable levels throughout the ectoderm (Figure 5D), rather than remaining localized to the dorsal midline as in control embryos (Figure 2A–2D). This expanded expression may imply an auto-activating circuitry of the Bmp synexpression group, as is known for *Drosophila* and vertebrates [90]. Then we assayed five genes that, in normal embryos, are expressed on or near the dorsal midline in either the ectoderm or endoderm, as candidates for activation by Bmp. *Dlx*, as described earlier (Figure 3A and 3B), is expressed in the ectoderm of the entire prosome [45] and on the dorsal midline of the meso- and metasome. In Bmp-treated embryos, *dlx* expression expands into all meso- and metasomal ectoderm (Figure 5I). A similar Bmp dependence is found for *tbx2/3,* a gene also normally expressed on the dorsal midline (Figure 3C and 3D). In Bmp-treated embryos, *tbx2/3* is expressed around the entire circumference of the embryo (Figure 5J). In the case of *pitx,* which has two ectodermal domains normally, the dorsal spot at the base of the proboscis expands in the presence of excess Bmp to encircle the base of the proboscis, along with underlying mesenchyme expression, as if repressed by Bmp. As further evidence of repression, *chordin* expression could not be detected in Bmp-treated embryos (Figures 5E and F).

Then we examined three genes normally expressed asymmetrically in the endoderm. *Hex* is normally expressed along the dorsal midline, with stronger expression anteriorly (Figures 3E and 5). After Bmp ligand treatment, this domain is expanded circumferentially most prominently in the anterior endoderm, but also at lower levels in posterior endoderm. Expression seems entirely down-regulated in the mid endoderm region, possibly at the level where gill slits would normally begin to develop (Figure 5M). *Pax 1/9* is normally expressed as a bilateral pair of spots in the dorsolateral endoderm as the first gill slit pair forms [45,91]. In treated embryos, these spots expand to a ring of expression around the body (Figure 5N). Similar results were also found with *nk2.3/2.5,* normally expressed on the dorsal midline of the endoderm, including the dorsal and medial region of the first gill slit pair. Like *pax1/9, nk2.3/2.5* expression expands to a ring encircling the entire endoderm (Figure 5O). As a final case, we chose *admp* as a candidate for Bmp repression since this gene is normally expressed only on the ventral midline of the ectoderm and endoderm. In Bmp-treated embryos, *admp* expression is indeed repressed, except for a residual small spot in the anterior endoderm (Figure 5P). Thus, uniform exposure of the embryo to excess Bmp protein activates the widespread expression of those genes normally expressed dorsally, and represses those normally expressed ventrally. These findings implicate dorsal midline Bmp in normal dorsoventral patterning in at least the ectoderm and endoderm.

We might expect very different effects of Bmp in specifying the nervous system of hemichordates. In *Drosophila* and vertebrates, Bmp represses neurogenesis, but in hemichordates there is no apparent segregation of ectoderm into neurogenic and epidermal territories. To test this, we examined the expression of *elv,* a pan neural gene [45]. In control embryos, *elv* is expressed throughout the ectoderm with its highest levels on the dorsal and ventral midlines and in the developing proboscis ectoderm (Figure 5G). In Bmp protein-exposed embryos, the expression of *elv* is not down-regulated; it is similar to controls along the anteroposterior axis and is expressed at uniform high levels around the embryo's circumference (Figure 5G and 5H). Therefore, unlike insects and vertebrates, hemichordates do not have a gene regulatory circuit by which Bmp represses neural fates during early development.

### Knockdown of Bmp by Small Interfering RNA

To further test the role of Bmp in dorsoventral patterning, we knocked down endogenous Bmp levels by injecting into the newly fertilized egg either a pool of small interfering RNA (siRNA) generated by the cleavage of the full-length transcript by the dicer enzyme or individual synthetic siRNAs directed against two different regions of the open reading frame of the *bmp2/4* mRNA. The phenotypes of the resulting embryos are identical and highly reproducible from all three kinds of materials and between injection runs. The embryos look normal throughout early stages; however, after gastrulation they fail to extend dorsally and remain cylindrical in organization, as we might expect from ventralized embryos. Anteroposterior organization is normal, as reflected by the fact that the prosome, mesosome, and metasome are set off by anterior and posterior grooves. On day 3, when the anterior groove would normally deepen on the ventral side to form the mouth (Figure 6A and 6B), the groove indents circumferentially and severs the mesosome from the prosome, which falls off (see Figure 6D–6F). Gill slits fail to form in the metasome. The telotroch remains close to the anus, and the posterior endoderm is wider than normal (Figure 6A and 6B). Overall, the effects of the siRNA injections to produce ventralized embryos complement those of Bmp ligand treatments to produce dorsalized embryos.

Indicative of the specificity of the anti-*bmp* siRNAs, control eggs injected with a scrambled siRNA sequence developed normally with a dorsoventral axis (unpublished data). Furthermore, when siRNA injected embryos were then treated with Bmp protein, they undertook Bmp-dependent gene expression, indicating that the siRNAs did not cause systemic damage unrelated to Bmp.

We examined the expression of four genes that normally have ventral domains and are therefore candidates for repression by endogenous Bmp from the dorsal side. As described earlier (Figure 3L and 3M), *netrin* is expressed in a broad band on the ventral side at early stages and then restricts down to a thin stripe on the ventral midline. In anti-*bmp* siRNA injected embryos, *netrin* expression expands uniformly throughout the ectoderm (Figure 6F), indeed suggesting that Bmp normally represses *netrin* dorsally. As a second example, *pitx* expression is altered in the embryos from eggs injected with anti-*bmp* siRNAs; the posterior ventral domain of *pitx* expands uniformly throughout the ectoderm (Figure 6D) whereas the anterior dorsal spot of expression is absent from the detached prosome. These data on *pitx* from Bmp knockdown embryos complement the results from the Bmp over-expression assays and suggest strongly that Bmp normally represses the expression of *pitx* to a posterior ventral stripe in the metasome but activates its expression in the anterior dorsal spot on the proboscis. Third, we examined *admp,* which is normally expressed in a ventral stripe. In anti-*bmp* siRNA embryos, *admp* expression expands uniformly, especially in the proboscis where it becomes very intense (Figure 6E). And fourth, in anti-*bmp* siRNA embryos, *dlx* expression in the prosome ectoderm remains patchy; however, the dorsal midline expression is absent (Figure 6C). Thus, Bmp is likely required for activation of *dlx* on the dorsal midline but not for prosome ectoderm expression.

In summary, Bmp2/4 activates and represses specific genes in the ectoderm, mesoderm, and endoderm germ layers to give unique dorsoventral patterns of expression in the embryonic body. Differences of Bmp availability along this axis are presumably related not only to the location of a Bmp source at the dorsal midline of the embryo and a Chordin (and perhaps Admp) source at the opposite midline, but also to the interactions of Bmp with a variety of modifiers encoded by genes of the Bmp synexpression group.

## Discussion

The anatomical components of the dorsoventral axis of hemichordates have been recognized for decades, such as the opposed midline axon tracts, giant neurons [92], the heart/kidney complex [64], gill slits, gonads, body wall muscle, and tail [93,94]. However, the homology of these to candidate counterparts in chordates has been ambiguous, making problematic the comparisons along the dorsoventral axes of the two groups. Molecular and developmental comparisons have been impossible given the paucity of data from hemichordates. We have improved the basis for comparison by supplementing anatomical observations with molecular data. In particular, our finding of the polarized expression of *bmp* and *chordin* on the two midlines of the early embryo establishes the existence of a Bmp-Chordin axis to which we can relate the locations of various gene expression domains and anatomical specializations of the definitive dorsoventral axis of the animal. These midlines seem to be the only sites of *bmp* and *chordin* expression in the embryo and juvenile. Furthermore, we have tested the developmental importance of the Bmp-Chordin axis and have found that at least 13 candidate target genes, which normally have dorsal or ventral expression, predictably expand or contract their domains in line with Bmp availability, as do all the observable anatomical specializations. At uniform high levels of exogenous Bmp we obtain cylindrical dorsalized embryos, and in the absence of endogenous Bmp we obtain ventralized embryos. All aspects of dorsoventral organization of ectoderm and endoderm, and likely also mesoderm (for which we lack good marker genes), depend on the Bmp-Chordin axis in the S. kowalevski embryo. As concluded previously for chordates and arthropods, and now supported by the hemichordate findings, this Bmp-Chordin axis is truly a developmental axis underlying the definitive anatomical dorsoventral axis, and it may date back to the earliest bilateral animals [1,27].

Furthermore, for comparisons with chordates, the Bmp-Chordin developmental axis of hemichordates can be defined intrinsically, without reference to unreliable features such as the orientation of the animal in the gravitational field or by the location of the mouth, features which have proven hard to compare across taxa. The expression of *bmp* mRNA at one pole of the Bmp-Chordin axis and *chordin* at the other suggests that a gradient of active Bmp protein across the embryo, high at the *bmp* pole and low at *chordin,* sets the quantitative conditions for selecting and organizing target gene expression. However, many modulators are known to affect the local level of active Bmp, such as the complexes it forms with Chordin, Tsg, and perhaps Cv-2, and its release from those complexes by the Tolloid protease [95]. The Bmp midline is also the site of expression of *bambi,* encoding a pseudoreceptor of Bmp which is thought to inhibit Bmp auto-activation [33] Furthermore, *admp,* which is expressed on the ventral midline, encodes a protein with Bmp-like action when released by Tolloid from its complex with Chordin, and then Admp acts through a receptor not used by Bmps [44]. Also, Bmp antagonists exist besides Chordin, such as Noggin and DAN. Preliminary tests indicate that these are widespread in the ectoderm of S*. kowalevskii,* not concentrated at a midline (unpublished data). Thus, the concentration profile of active Bmp protein across the embryo may not be a monotonic gradient. Still, whatever its shape, we propose that the Bmp profile constitutes the fundamental developmental axis for the selection and placement of components of the eventual anatomical dorsoventral axis.

While the Bmp-Chordin axis is shared by chordates and hemichordates, it remains to compare what target genes and anatomical specializations are indeed selected and placed on the axis in the two groups. On the Bmp side, both groups place gill slits, coelomic mesoderm, a contractile heart-like dorsal vessel, and *nk2.3/2.5* expression; on the Chordin side, both groups place the post-anal tail, the majority of striated body wall muscle and motor neurons, and *netrin1* expression. These shared features, including their similar placements along the Bmp-Chordin axis, can be ascribed to the deuterostome ancestor, and some perhaps even to the bilateral ancestor. These features were just brought forward in the chordate lineage (hemichordate, too), not evolved anew within the lineage.

There are two prominent exceptions to the list of targets of the Bmp-Chordin axis shared by hemichordates and chordates, namely, the nervous system and the mouth. The hemichordate diffuse nervous system does not reveal a dorsoventral asymmetry of neurogenesis or neuron abundance; it develops throughout the ectoderm, even at the midline of *bmp* expression. In chordates (and *Drosophila,* too) the central nervous system develops near the source of Chordin, and the large epidermal territory develops near the Bmp source. In hemichordates, neurogenic and epidermal cells are finely intermixed throughout the ectoderm [21]. In chordates and *Drosophila,* increased Bmp contracts the developing territory of neurogenic ectoderm, and expands the epidermal territory, whereas decreased Bmp results in the reverse effect [90]. However, as shown here, the exposure of the hemichordate embryo to a uniform excess of exogenous Bmp protein, which up-regulates endogenous *bmp* gene expression around the body, does not cause the territory of neurogenesis to contract, as shown by the persistence of widespread pan-neural gene expression (Figure 5H). This insensitivity of neurogenesis to Bmp in hemichordates, despite the presence of a Bmp-Chordin axis in the embryo, implies that the “anti-neurogenic phase” of Bmp signaling is absent in hemichordates.

Nonetheless hemichordates use the Bmp-Chordin axis for a second phase, the “morphogenetic phase” for patterning the three germ layers. The patterning of neuronal cell types within the diffuse nervous system is included in the patterning, for example, the giant neurons near the dorsal midline (the Bmp pole); a nerve tract rich in motor neuron axons at the ventral midline, a specialized patch of sensory neurons near the mouth, and a line of *poxN* expressing cells, perhaps specialized neurons, on the posterior dorsal midline. However, chordates appear to achieve much more patterning of neuronal cell types than do hemichordates. Indeed, the entire Bmp-Hh double gradient used by chordates to select and place ten gene expression domains in the dorsoventral axis of the neural tube [96], is absent from hemichordates. Hh is localized to the apical ectoderm and anterior gut in hemichordates; it has no dorsoventral expression. Whereas the neural tube domains of chordates include those of *pax6, dbx, en, irx, nk2.2,* and *msx,* none of these genes shows a dorsoventral domain in S. kowalevskii (see Results here and in [45]). Yet all of these are expressed in the hemichordate embryo in anteroposterior domains, as they are in chordates in domains additional to the dorsoventral domains. Particularly noteworthy are the *nk2.2* and *msx* domains since these are thought to have similar expression in *Drosophila* neurectoderm and the chordate neural tube [15]. Yet they have no dorsoventral domains in *S. kowalevskii.* These differences suggest that much of the regulatory architecture involved in dorsoventral patterning of chordate nervous system evolved subsequent to the divergence of hemichordates and chordates. If true, the dorsoventral axis would have been a locus of much more evolution in chordates than was the anteroposterior axis since, as we showed previously [45], the gene expression domains of this axis are extensively similar in both groups, hence in the deuterostome ancestor.

Further analysis will be needed to determine if the neural default circuitry of the kind operating in vertebrates [97] is entirely absent from hemichordates. If so, the circuitry and its role in segregating neural and epidermal territories must have arisen independently in the chordate and arthropod lines, and perhaps in other lines as well [19]. Such segregation was presumably a key evolutionary step in the centralization of the nervous system, which is a major dorsoventral innovation. Presumably, intrinsic to the evolution of a restriction of neural and epidermal fates in the different lineages was the use in all lineages of the pre-existing Bmp-Chordin axis. Alternatively, hemichordates (and perhaps echinoderms) may have lost the central nervous system of a deuterostome ancestor, rendering it diffuse and presumably simplified. However, we think this unlikely because the domain map of anteroposterior neural patterning genes of hemichordates does not seem to be reduced in complexity relative to chordates [45] and because the germ layers of hemichordates seem otherwise rich in dorsoventral differentiations, except for neural-epidermal segregation.

In the context of the Bmp-Chordin axis, we must last discuss the location of the mouth in hemichordates. It is on the Chordin side, as in *Drosophila,* but in chordates it is on the Bmp side. Chordates then, among protostomes and deuterostomes, would be the only group to have this mouth location, a conclusion reached by Nubler-Jung and Arendt [25] from anatomical comparisons, now reinforced by our elucidation of the Bmp-Chordin axis in hemichordates. The recent finding of left-sided expression of *pitx* and *nodal* in sea urchin larvae has been presented as evidence that echinoderms are also uninverted [59], although direct comparisons of larval and adult body plans are problematic. Wherever the mouth is, that side is called ventral, by convention, thereby fixing the orientation of the anatomical dorsoventral axis. The inverse relation of the axes of chordates and arthropods was furthermore recognized by the positions of heart, coelomic mesoderm, body wall muscle, and nerve cords. Thus, the dorsoventral axis of chordates is the inverse of all other bilateral animals.

**Figure 7:**
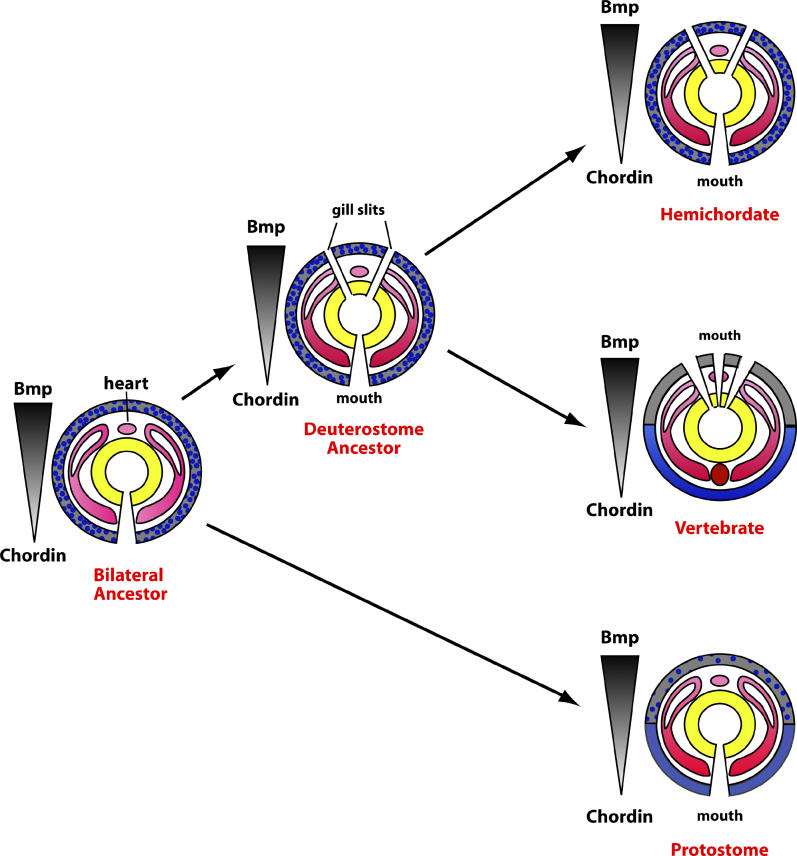
Summary of Inferred Evolutionary Changes of the Dorsoventral Axis in Deuterostome Evolution, with Emphasis on Hemichordates and Chordates Mesoderm is shown in red, endoderm in yellow and ectoderm in blue (neural) and grey (epidermis). An ancestral secreted Bmp axis was involved in dorsoventral patterning of the three germ layers in the bilaterian ancestor. This ancestor we propose was characterized by a diffuse organization of its nervous system, shown by blue dots. A Bmp gradient was involved in dorsoventral patterning of all three germ layers. In the basal deuterostome and hemichordates the role of the Bmp gradient is conserved in general dorsoventral patterning. During chordate (and protostome) evolution, the existing Bmp/Chordin axis was co-opted for an additional developmental role in nervous system centralization.

A venerable explanation of the inverse relationship is the inversion hypothesis (dating back to E. Geoffroy Saint-Hilaire [26]) in which a chordate ancestor inverted its entire body dorsoventrally and then moved the mouth to the opposite side (or the reverse order of events). Our current studies of altering Bmp levels in development indicate that mouth development is repressed by Bmp and hence it arises at the point farthest from Bmp, in the mesosome-metasome groove. Bmp-overexposed embryos lack a mouth, and Bmp-deficient embryos form a mouth circumferentially which causes the proboscis to fall off (Figures 5 and 6). The new mouth of chordates would seem to hold a new relationship to the Bmp-Chordin axis. The classical inversion hypothesis, despite its attractions, does not require the nervous system to have been already centralized before inversion occurred, even though such centralization is usually assumed. Our data raise the possibility that centralization and inversion are separate issues (outlined in Figure 7). The ancestor, even with a diffuse system, could have inverted its body, which already had dorsoventral differences in the other germ layers, and then moved its mouth. Subsequently it could have centralized its nervous system to the Chordin side. Such a scenario works just as well, indicating that inversion can't be used to argue the existence of a central nervous system in the uninverted deuterostome or bilateral ancestor.

However, there are other ways to achieve inversion in chordates, other than assuming a major shift in the life history mode of an ancestor. One of the ways might be termed the “ectopic chordin hypothesis.” In chordates, Chordin is produced by both endomesoderm of Spemann's organizer, and the neurogenic region of the ectodermal blastula stage, at least in Xenopus laevis [42] whereas in all other phyla, including hemichordates, the source of Chordin is exclusively the ectoderm. As shown here for hemichordates and elsewhere for other bilateral animals, all organs, tissues, and expression domains of the definitive dorsoventral axis depend on the Bmp-Chordin axis for their selection and placement. If this developmental axis were inverted during very early development, without moving the mouth-forming agency, all aspects of definitive dorsoventral organization would necessarily invert. In chordates, Bmp is produced “at large” in the ectoderm whereas the Chordin-Admp source is primarily localized in the chordamesoderm which arises from the archenteron/gut midline. Wherever the Chordin-Admp source is placed, this defines the Bmp-Chordin axis in chordates. Perhaps this new germ layer source, so different from ectoderm, evolved on the side opposite the mouth in a chordate ancestor, thus inverting the axis. To further these inquiries, we plan to examine the means by which the Bmp and Chordin stripes are placed opposite one another in the ectoderm of the hemichordate embryo, and the means of placement of the mouth.

Whatever the means by which the mouth and the central nervous system are produced in chordates, these studies reinforce the prominence of the Bmp-Chordin axis as a patterning mechanism in perhaps all bilaterian animals. The hemichordate S. kowalevskii seems to be an exceptional object for the study of the origin of the chordate body plan. This is in part because of the functional independence of the dorsal-ventral and anterior-posterior axes and the fact that patterning genes often appear dedicated to a single use and not used successively in new patterns. From our studies we can conclude that in the deuterostome lineage, the anteroposterior pattern, delineated by *hox* and other genes, may have been established very early, as a modest but significant modification of the bilaterian ancestor. In the dorsoventral dimension, the basic Bmp-Chordin axis would have been just as fundamental in the common lineage of bilaterians, but considerable modification of the subdivisions of this axis has certainly occurred in deuterostomes. The centralized nervous system of arthropods and vertebrates, so important in the previous theories of anatomical evolution of the bilateral organisms, may itself have arisen independently in both groups but built on the pre-existing Bmp-Chordin gradient. The studies in hemichordates suggest strongly that the chordate body plan emerged from the deuterostome ancestor's body plan by maintaining the anteroposterior patterning and the Bmp-Chordin gradient, but re-specifying features such as the mouth, the nervous system, and numerous details of dorsoventral patterning.

## Materials and Methods

### Eggs, embryos, and juveniles.

Adult S. kowalevskii were collected intertidally in September near Woods Hole, Massachusetts. Ovulation and fertilization were achieved in the laboratory by the methods of Colwin and Colwin [98,99] with several modifications [91]. Embryos were staged by the normal tables of Bateson [100–102] and Colwin and Colwin [103].

### Library construction.

Two cDNA libraries were used in this study, one from mixed blastula and gastrula stages and another from mixed gastrula and neurula stages. These were constructed as described in Lowe et al. [45].

### Cloning of orthologs.

Three strategies were used: 1) We surveyed 60,000 arrayed expressed sequence tag clones from the two libraries. 2) We screened cDNA libraries at low stringency using short probes complementary to highly conserved regions of orthologs from other deuterostomes using standard protocols. 3) We designed degenerate primers (Codehop http://blocks.fhcrc.org/codehop.html) for PCR assay of ortholog sequences in arrayed aliquots of the cDNA libraries.

### Functional assays.

Over-expression of Bmp2/4 was achieved by addition of recombinant Zebrafish Bmp4 protein (R&D Systems, Minneapolis, Minnesota, United States) at four concentrations (10, 100, 250, and 500 ng/ml) from the blastula stage until day 4 of development. Ligand protein was replaced every 24 h of the treatment. Samples were fixed in MEMFA and stored in ethanol [91]. For the depletion of Bmp2/4, we injected siRNA. Two different strategies were carried out: 1) Double stranded transcripts of 800 bp in length were produced which were then digested with dicer enzyme (Gene Therapy Systems, San Diego, California, United States) according to the manufacturers protocols. The stock solution for injection was 50 ng/ul in water. 2) We designed synthetic siRNAs to two regions of the *bmp2/4* open reading frame. In one case, the sequences were: sense 5′-CUACGGACUCGAAGUGGAAUU-3′ and antisense 3′-UUGAUGCCUGAGCUUCACCUU-5′ which were directed to region 1057–1077 of the *bmp2/4* clone. In the other case, the sequences were: sense: 5′-CUCGACCAAUCAUGCGAUAUU-3′ and antisense: 3′-UUGAGCUGGUUAGUACGCUAU-5′ which were directed to positions 1396–1416. The stock solutions for injection were 50 ng/ul in water. For injection of the siRNAs, several eggs were added to a small dish containing freshly diluted sperm for 15 s and then transferred to a silicon rubber injection chamber. A quantitative injection method with front loaded needles was used [104]. The injections were done under an upright microscope with a 10× 0.3 NA objective lens. The 360 μm diameter egg has a volume of approximately 25 nl, and each egg was injected with 0.23 nl of siRNA solution (50 pg/nl stock or a dilution thereof), that is, less than 1% volume. The optimal period for injection was from approximately 3 min to 6 min after fertilization. At other times, the eggs usually lysed while using this injection method. Successfully injected embryos were cultured in sterile seawater in 35 mm culture dishes (coated with agarose to reduce sticking).

### In situ hybridization.

This was done as described in Lowe et al. [91]. After staining, samples were fixed overnight in Bouins fixative and then rinsed multiple times in 80% EtOH/ 0.1M TrisHCl [pH 8] until the picric acid color was gone. Samples were then further dehydrated into 100% methanol and cleared in Murray clear (benzyl benzoate: benzaldehyde, 2:1) and mounted onto slides in Permount (Fisher Scientific, Pittsburgh, Pennsylvania, United States).

### Gene orthology and phylogenetic analysis.

All genes of this study were assigned orthology by phylogenetic analysis. The results are presented in Figures S1–S4. Top amino acid blast hits were aligned with additional sequences in ClustalX (http://www.embl.de/~chenna/clustal/darwin/index.html). Neighbor-joining phylogenetic analyses were carried out in PAUP* (http://paup.csit.fsu.edu) restricting the analysis to conserved domains from the alignments. 1,000 bootstrap replicates were carried out, and nodes with less than 50% support were collapsed. Bayesian analysis was carried out using Mr Bayes (v3.0B4) (http://mrbayes.csit.fsu.edu). The parameters of the analysis are as follows: 10,000,000 generations, burn in 7,500, sampling frequency 100, and four chains, under a mixed model of evolution.

## Supporting Information

Figure S1 Gene Trees for Study Genes (A) Transforming growth factor class genes *admp, bmp2/4,* and *bmp5/8,* (B) *tsg,* (C) *cv2,* (D) *tbx2/3.*
(8.2 MB TIF)

Figure S2 Gene Trees for Study Genes (E) *pitx,* (F) *hex,* (G) *olig,* (H) *pox neuro.*
(8.2 MB TIF)

Figure S3 Gene Trees for Study Genes (I) *lim3,* (J) *sim,* (K) *netrin1/2,* (L) *mox.*
(8.2 MB TIF)

Figure S4 Gene Trees for Study Genes (M) *mnx,* (N) *hh,* (O) *msx,* (P) *nk2.3/2.5.*
(8.2 MB TIF)

### Accession Numbers

The GenBank (http://www.ncbi.nlm.nih.gov/Genbank/index.html) accession numbers for the genes and gene products discussed in this paper are *admp* (DQ431039), *bambi* (DQ431032), *bmp2/4* (DQ431030), *bmp5/8* (DQ431031), *chordin* (DQ431034), *cv* (DQ431033), *dlx*(AY318740.1), *hh*(DQ431035), *hex* (DQ431047)*, lim3/4* (DQ431040), *mnx* (DQ431042), *mox* (DQ431050), *msx* (DQ431048), *netrin1/2* (DQ431045), *nk2–2* (DQ431049), *nk2–3/2–5* (DQ431046), *olig* (DQ431044), *pax1/9* (DQ869011), *pitx* (DQ431041), *pox-neuro* (DQ431038), *sim* (DQ431043), *tbx2/3* (DQ431037), *tolloid* (DQ431036), and *tsg* (DQ810291).

## Abbreviations

Admp: anti-dorsalizing morphogenetic protein
Bmp: bone morphogenetic protein
Cv: crossveinless
*dlx*: *distalless*
Hh: hedgehog
*pitx*: *pituitary homeobox*
PoxN: pox neuro
RNAi: RNA interference
Shh: sonic hedgehog
Sim: single-minded
siRNA: small interfering RNA
Tsg: twisted gastrulation
