# Correction: Dorsoventral Patterning in Hemichordates: Insights into Early Chordate Evolution

**DOI:** 10.1371/journal.pbio.1002354

**Published:** 2015-12-29

**Authors:** Christopher J Lowe, Mark Terasaki, Michael Wu, Robert M Freeman, Linda Runft, Kristen Kwan, Saori Haigo, Jochanan Aronowicz, Eric Lander, Chris Gruber, Mark Smith, Marc Kirschner, John Gerhart

The in situ staining patterns of Fig 4C and 4D were incorrectly assigned to the *nkx2*.*2* gene of *Saccoglossus kowalevskii*. Subsequent sequencing and analysis has shown that these images were actually expression patterns for the *foxA* gene. Fig 4 has been updated with images of the in situ staining patterns of the *nkx2*.*2* gene of *Saccoglossus kowalevskii* and the figure legend has been amended to reflect this.

The corrected legend for Fig 4 appears below.

Additionally, the authors would like to amend the text description of *nk2-2* expression on p.1610 (right hand column), which incorrectly concerned *foxa*; and restate the conclusion about *nk2-2* expression in the Discussion on p.1615 (left column). The authors confirm this conclusion is unchanged, but the old *foxa* data were irrelevant to the cross-phylum comparison of *nk2-2* expression among *S*. *kowalevskii*, chordates, and *Drosophila*.

The corrected text appears below.

Page 1610:

In *S*. *kowalevskii*, we find that the expression profiles of the *hh*, *nk2*.*2*, and *msx* orthologs do not at all resemble the expression domains of vertebrates or *Drosophila*, as shown in Fig 4. We could isolate only one kind of hedgehog ortholog from *S*. *kowalevskii*, but this may exhaust the hemichordate repertoire since basal deuterostomes such as amphioxus and sea urchins have but one gene [86,87], whereas vertebrates have up to four paralogs of *hh* [86]. The expression of *hh* in *S*. *kowalevskii* begins at day 2, in a small patch at the apical tip of the prosome ectoderm (Fig 4A), and it continues in the same domain throughout all stages examined (Fig 4B). Low level expression also occurs in the anterior gut (unpublished data). At no time is *hh* expressed in a dorsal or ventral midline domain, for example, close to the netrin ventral domain. The *nk2-2* gene (also called *nk2*.*2* and *nkx2*.*2*) of *S*. *kowalevskii* was cloned by low stringency hybridization. During gastrulation, it is expressed in the archenteron endoderm (Fig 4C) rather than ectoderm, as has also been found in amphioxus, though not other chordates [88]. Expression in *S*. *kowalevskii* is excluded from the anterior mesendoderm that becomes the prosome mesoderm, and the level of expression is reduced in the posterior endoderm. During days 2 and 3 of development, *nk2-2* expression is further down-regulated in in the posterior and midline endoderm, leaving by day 3.5 (Fig 4D) a left and right patch of expression in the pharyngeal endoderm, ventral to the site of gill pore formation. At no stage was *nk2-2* expressed in an ectodermal dorsal or ventral midline domain. Thus, like *hh* of *S*. *kowalevksii*, the *nk2-2* gene, which in chordates depends on Hh signaling for expression, is not expressed in a chordate-like ectodermal midline domain.

Page 1615:

Nonetheless hemichordates use the Bmp-Chordin axis for a second phase, the “morphogenetic phase” for patterning the three germ layers. The patterning of neuronal cell types within the diffuse nervous system is included in the patterning, for example, the giant neurons near the dorsal midline (the Bmp pole); a nerve tract rich in motor neuron axons at the ventral midline, a specialized patch of sensory neurons near the mouth, and a line of *poxN* expressing cells, perhaps specialized neurons, on the posterior dorsal midline. However, chordates appear to achieve much more patterning of neuronal cell types than do hemichordates. Indeed, the entire Bmp-Hh double gradient used by chordates to select and place ten gene expression domains in the dorsoventral axis of the neural tube [96] is absent from hemichordates. Hh is localized to the apical ectoderm and anterior gut in hemichordates; it has no dorsoventral expression. Whereas the neural tube domains of chordates include those of *pax6*, *dbx*, *en*, *irx*, *nk2*.*2*, and *msx*, none of these genes shows a dorsoventral ectodermal midline domain in *S*. *kowalevskii* (see Results here and in [45]). Yet all of these are expressed in the hemichordate embryo in anteroposterior domains, as they are in chordates in domains additional to the dorsoventral domains. Particularly noteworthy are the *nk2*.*2* and *msx* domains since these are thought to have similar expression in *Drosophila* neurectoderm and the chordate neural ectoderm of the neural tube [15], with roles in neural patterning. Yet neither gene has a dorsal or ventral ectodermal domain in *S*. *kowalevskii*. These differences suggest that much of the regulatory architecture involved in dorsoventral patterning of chordate nervous system evolved subsequent to the divergence of hemichordates and chordates. If true, the dorsoventral axis would have been a locus of much more evolution in chordates than was the anteroposterior axis since, as we showed previously [45], the gene expression domains of this axis are extensively similar in both groups, hence in the deuterostome ancestor.

**Fig 4:**
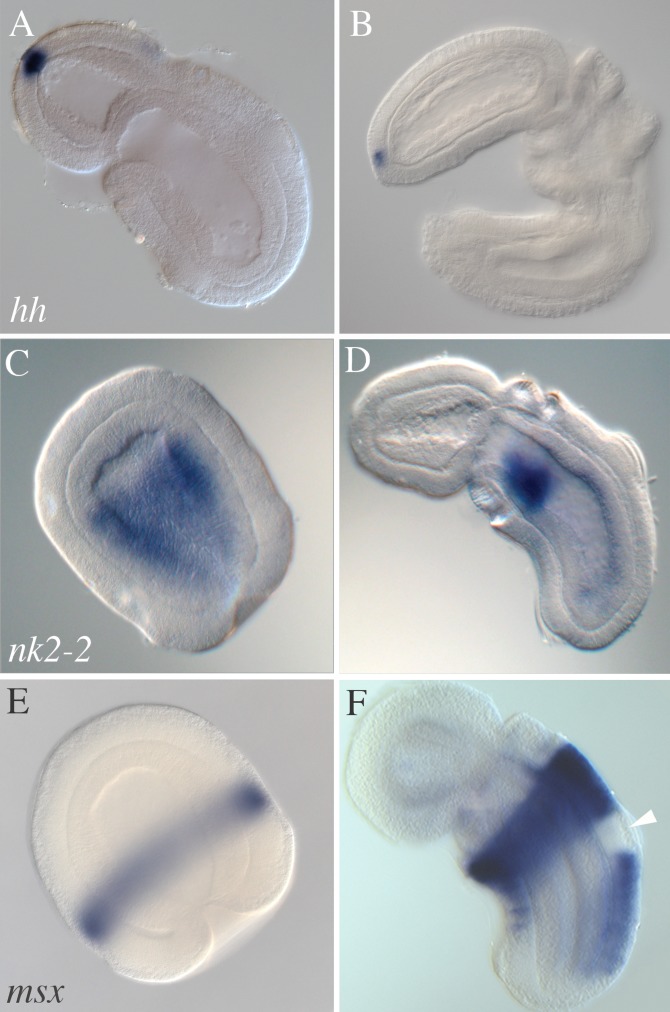
Expression in *S*. *kowalevskii* of Orthologs of Chordate Genes Important in Dorsoventral Patterning of the Neural Tube. All embryos are shown as optical sections, and oriented in a similar manner as in Figure 2 with anterior to the top and left of each panel and dorsal in the top right of each panel, unless otherwise specified. (A) *Hh* expression in the apical tip of a day 3 embryo, and (B) at day 5 at the same location. (C) Endodermal expression of *nk2–2* (also called *nkx2*.*2*) in the late gastrula, with a sharp delineation between presumptive proboscis mesoderm and definitive endoderm, and with reduced expression in the posterior endoderm, and (D) endodermal expression of *nk2-2* at day 3.5, with left-right lateral patches in the pharyngeal endoderm ventral to the site of gill pore formation and with expression down-regulated in other regions of the endoderm. (E) Expression of *msx* in the late gastrula, and (F) at day 3. Expression occurs exclusively in the ectoderm of the metasome and is down-regulated in the telotroch, the cilated band, as marked (white arrowhead).
